# P-1527. Update on the Activity of Ceftobiprole against Gram-positive Clinical Bacterial Isolates Causing Skin and Skin-structure Infections in the United States (2016–2020)

**DOI:** 10.1093/ofid/ofae631.1696

**Published:** 2025-01-29

**Authors:** Rodrigo E Mendes, Joshua Maher, Hank Kimbrough, Jennifer Smart, Mark E Jones, Mariana Castanheira, Helio S Sader

**Affiliations:** JMI Laboratories, North Liberty, Iowa; Element Materials Technology/Jones Microbiology Institute, North Liberty, Iowa; Element Materials Technology/Jones Microbiology Institute, North Liberty, Iowa; Basilea Pharmaceutica International Ltd, Allschwil, Basel-Landschaft, Switzerland; Basilea Pharmaceutica International Ltd., Allschwil, Switzerland, Allschwil, Basel-Landschaft, Switzerland; JMI Laboratories, North Liberty, Iowa; JMI Laboratories, North Liberty, Iowa

## Abstract

**Background:**

Ceftobiprole (BPR) is a fifth-generation cephalosporin with broad spectrum activity against both Gram-negative and -positive (GP) bacteria, including methicillin-resistant *Staphylococcus aureus* (MRSA). BPR was approved for treatment of community- and hospital-acquired pneumonia in Europe and other regions. This agent was recently approved by the US-FDA for treatment of adult *S. aureus* bacteremia, including right-sided infective endocarditis, acute bacterial skin and skin structure infections (ABSSSIs) in adults, and adult and pediatric community-acquired bacterial pneumonia. This study assessed the *in vitro* activity of BPR and comparator agents against contemporary clinical isolates causing SSSIs in the US.

**Methods:**

Between 2016–2020, the SENTRY Antimicrobial Surveillance Program collected over 12,200 isolates causing SSSI in patients at 33 US medical centers. Isolates were tested for susceptibility (S) by broth microdilution according to CLSI reference methodology with CLSI, FDA, and EUCAST interpretive criteria applied, where applicable.

**Results:**

Evaluated SSSI causing GP bacteria (*n*=8,120) comprised primarily of the following species: *S. aureus* (6,406, 78.9%), beta-hemolytic streptococci (BHS; 741, 9.1%), and *Enterococcus faecalis* (401, 4.94%). BPR was active against *S. aureus* (MIC_50/90_, 0.5/1 mg/L; 99.8% S by EUCAST and FDA criteria). Against MRSA (*n*=2,729) and MSSA (*n*=3,677) isolates, BPR maintained activity with 99.5% MRSA and 100% MSSA inhibited at the FDA/EUCAST S breakpoint of ≤2 mg/L. BPR displayed activity against other GP species groups, including BHS (MIC_50/90_, 0.015/0.03 mg/L), Viridans group streptococci (MIC_50/90_, 0.06/0.12 mg/L) and *E. faecalis* (MIC_50/90_ 0.5/2 mg/L). BPR also displayed activity against resistant subsets, such as clindamycin-R MRSA, tetracycline-R MRSA, and vancomycin-R *E. faecalis* (MIC_50/90_, 1-2/2 mg/L).

**Conclusion:**

BPR showed potent *in vitro* activity against clinical GP bacterial isolates from major SSSI pathogen groups causing SSSI in US hospitals. In addition, BPR sustained activity when tested against antibiotic-resistant subsets. With the recent FDA approvals for various serious bacterial infections, BPR becomes an additional option to treat SSSI in the US.

**Disclosures:**

**Rodrigo E. Mendes, PhD**, GSK: Grant/Research Support **Jennifer Smart, PhD**, Basilea Pharmaceutica International Ltd: Employee|Basilea Pharmaceutica International Ltd: Stocks/Bonds (Public Company) **Mark E. Jones, PhD**, Basilea Pharmaceutica International Ltd: Employee|Basilea Pharmaceutica International Ltd: Stocks/Bonds (Public Company)

